# Bayesian panel smooth transition model with spatial correlation

**DOI:** 10.1371/journal.pone.0211467

**Published:** 2019-03-04

**Authors:** Kunming Li, Liting Fang, Tao Lu

**Affiliations:** 1 College of Economics, Fujian Agriculture and Forestry University, Fuzhou, Fujian, China; 2 School of Economics & Management, Fuzhou University, Fuzhou, Fujian, China; 3 Department of Mathematics and Statistics, University of Nevada, Reno, Nevada, United States of America; Beijing University of Technology, CHINA

## Abstract

In this paper, we propose a spatial lag panel smoothing transition regression (SLPSTR) model ty considering spatial correlation of dependent variable in panel smooth transition regression model. This model combines advantages of both smooth transition model and spatial econometric model and can be used to deal with panel data with wide range of heterogeneity and cross-section correlation simultaneously. We also propose a Bayesian estimation approach in which the Metropolis-Hastings algorithm and the method of Gibbs are used for sampling design for SLPSTR model. A simulation study and a real data study are conducted to investigate the performance of the proposed model and the Bayesian estimation approach in practice. The results indicate that our theoretical method is applicable to spatial data with a wide range of spatial structures under finite sample.

## Introduction

In panel data regression models, cross sectional and time effects are usually introduced to represent individual heterogeneity. And the coefficients of explanatory variables are assumed to be constant for all section units and periods. In practice, this assumption is sometimes unreasonable. For example, many empirical studies have found that, the impact of exchange rate fluctuation on domestic inflation is not the same in countries with different degree of openness. At this point, if we use traditional panel data models to study the relationship between exchange rate and inflation based on data of different countries, this is equivalent to imposing the assumption that exchange rate has the same effect on inflation in all countries., which is somewhat far-fetched In order to overcome this drawback of traditional panel data models, economists propose random coefficients panel data models and varying coefficients panel data models in which the coefficients can vary with section units and times. The panel data threshold regression model (PTR) proposed by [1] is a widely used varying coefficients model in which the coefficients vary when threshold variables are in different threshold intervals that represent several regimes. Therefore, the coefficients are time-varying if threshold variables change over time. However, the changes in coefficients of PTR model are discontinuous in general since there are usually only a limited number of threshold intervals, which indicates that the transition between different regimes is abrupt. This limits the application scope of the model to a certain extent.. As an effective extension of the PTR model, the panel data smoothing transition regression model (PSTR) proposed by [2] and [3] allows coefficients to vary continuously with transition variables, which effectively ensures the continuity of regimes transition PSTR model actually allows coefficients to change with cross sections and times, which is a sufficient relaxation of heterogeneity assumption in panel data model, and we can easily find that the PTR model is a special case of the PSTR model. As individual heterogeneity can be fully portrayed in PSTR model, this model has been widely used in empirical research in many areas, such as [2], [4], and so on.

Traditional econometric models generally assume that dependent variables are uncorrelated among cross-sections, but more and more studies have found the existence of cross-sectional correlation. For example, the local equilibrium prices of all local markets in the general equilibrium model are correlated, the individuals in the network model are interconnected, and one participant’s decision is influenced by other participants’ in a competitive market, and so on. When facing the above research topics, the traditional econometric model will no longer be applicable. In contrast, the spatial econometric models can well deal with cross-section correlated problems. This characteristic makes this kind of model the standard analysis tool in the empirical research of social network, strategy interaction, peer effects and many other fields. The main advantage of PSTR model is that it can well handle cross-sectional and time heterogeneity. However, this model is not able to deal with spatial data with cross-section correlation. In order to combine the advantages of spatial model and PSTR model, this paper introduces spatial correlation into PSTR model and proposes a spatial lag panel smooth transition regression (SLPSTR) model which can fully consider heterogeneity and spatial correlation simultaneously.

Frequentist estimation methods are widely used in econometric literature. Scholars often use Instrumental Variable Method (IV), Generalized Moment Method (GMM) and (Quasi) Maximum Likelihood Method to estimate spatial econometric model and Nonlinear Least Squares Method to estimate PSTR model. Although the model (SLPSTR) studied in this paper is a combination of spatial econometric model and PSTR model, the estimation methods of these two models cannot be directly applied to SLPSTR model. We propose a Bayesian inference method for this model. Compared with frequentist method, the most important feature of Bayesian estimation is the use of information. The Bayesian method takes into account both sample information and priori information, while the frequentist method only uses sample information. Due to the use of more information, we can obtain more robust and accurate estimation results by Bayesian methods. With the development of computer technology and the deep research of Bayesian theory, more and more literature pay attention to the application of Bayesian method in spatial econometric models, among which, [5] and [6] studied Bayesian estimation of spatial autoregressive models and limited dependent variable spatial autoregressive models respectively. [7] proposed the Bayesian probit model with spatial dependence. [8] developed a spatial Dirichlet process model for spatial data and discussed its properties and Bayesian estimation. [9] studied the Bayesian estimation and model selection for spatial Durbin error model with finite distributed lags. In the study of panel data spatial econometric model, [10] proposed the Bayesian inference for the spatial random effects model. [11] introduced the Bayesian approach to analyze spatial econometric panel data mode. [12] studied spatial autoregressive models with unknown heteroskedasticity and made a comparison of Bayesian and robust GMM approach. Their results indicated that the Bayesian estimators perform better than the robust GMM estimator in terms of finite sample efficiency. The above existing studies can provide an important reference for this paper.

This paper can also have some contributions to the field of spatial econometrics. First of all, the existing literatures recently focus on the linear spatial model with the assumption that the influence of the independent variables on the dependent variable is linear and the marginal effects are constant in different time and space. Different from the above assumption, this paper introduces a nonlinear influence form of “regime transition” into spatial econometric model and gets the spatial panel smooth transition model which allows the influence of independent variables on the dependent variable to change with some transition variable. Since transition variable often changes over time and space, the influence of the independent variables can also be time and spatial-varying, which relaxes the assumption of linear model that the coefficients of independent variables are constant. At the same time, this paper also constructs a Bayesian inference method for the proposed model. The advantage of Bayesian method is that it comprehensively utilizes priori and posteriori information and thus has higher estimation accuracy and robustness. Therefore, our work not only expands the research field of spatial econometric models, but also provides a new analysis tool for application researchers, which also reflects the value and innovation of this paper.

The rest of the paper is arranged as follows: the second section introduces the model studied in this paper, the third section proposes a Bayesian estimation method for the model, the fourth and the fifth section are numerical simulation and real data example, respectively, the last section is the conclusion.

## Model

The spatial lag panel smooth transition regression (SLPSTR) model considered in this paper has the form as follows

$$y_{it}=\rho {\left(WY\right)}_{it}+{x^{\prime }}_{it}\beta_{0}+{x^{\prime }}_{it}\beta_{1}g(q_{it};\gamma ,c)+\mu_{i}+\varepsilon_{it},\;i=1,2,\cdots ,N;t=1,2,\cdots ,T$$ (1)

where the subscript *i*,*t* indicates *i-th* cross-section and *t-th* period respectively, *y*_*it*_ is dependent variable, **Y** = (*y*_11_,*y*_21_,⋯,*y*_*N*1_,*y*_12_,⋯,*y*_*NT*_)′ is *NT* × 1 vector of dependent variables and **W** is *NT* × *NT* spatial weight matrix, **x**_**it**_ is *k* × 1 vector of independent variables, **β**_0_,**β**_1_ are *k* × 1 vectors of coefficients, *μ*_*i*_ represents the individual fixed effects, *ε*_*it*_ is random error term and *ε*_*it*_ ∼ *N*(0,*σ*^*2*^), $g(q_{it};\gamma ,c)=[1+\text{exp}(-\gamma {\left(\prod_{j=1}^{m}\left(q_{it}-c_{j}))\right)\right]}^{-1}$ is transition function and evidently we have 0 < g(*q*_*it*_;*γ*,**c**) < 1, where **c** = (*c*_*1*_,*c*_*2*_,⋯,*c*_*m*_)′ is m × 1 vector of location parameters, *γ* > 0 is scale parameter. Without loss of generality, we set *m* = 1 to simplify mathematical deduction.

Given *i*, SLPSTR model can also be written as

$$Y_{i}=\rho {\left(WY\right)}_{i}+{X^{\prime }}_{i}\beta_{\text{0}}+G_{i}{X^{\prime }}_{i}\beta_{\text{1}}+\mu_{i}e+\varepsilon_{i}$$ (2)

where **Y**_*i*_ = (*y*_*i*1_,*y*_*i*2_,⋯,*y*_*iT*_)′, e = (1,1,⋯1)′ is a *T* × 1 vector with all elements valued 1, **X**_*i*_ = (*x*_*i*1_,*x*_*i*2_,⋯,*x*_*iT*_), G_*i*_ = *diag*(*g*(*q*_*i*1_;*γ*,**c**),⋯,g(*q*_*i*T_;*γ*,**c**)), and **ε**_*i*_ = (*ε*_*i*1_,*ε*_*i*2_,⋯,*ε*_*iT*_)′.,

Assuming that $Y=({Y^{\prime }}_{1},{Y^{\prime }}_{2},\cdots ,{Y^{\prime }}_{N}{)}^{\prime }$, **X** = (**X**_1_,**X**_2_,⋯,**X**_*N*_)′, $E=({E^{\prime }}_{1},{E^{\prime }}_{2},\cdots ,{E^{\prime }}_{N}{)}^{\prime }$, where E_i_ = (0,**e**,0) is a *T* × *N* matrix in which the elements of *i*-*th* column are one and other elements are all zero, **G** = *diag*(**G**_1_,**G**_2_,⋯,**G**_**N**_),**Z** = (**E**⋮**X**⋮**GX**),$\Theta =(\mu_{1},\mu_{2},\cdots ,\mu_{N},{\beta^{\prime }}_{0},{\beta^{\prime }}_{1}{)}^{\prime }$, and $\varepsilon =({\varepsilon^{\prime }}_{1},{\varepsilon^{\prime }}_{2},\cdots ,{\varepsilon^{\prime }}_{N}{)}^{\prime }$, then the two regimes SLPSTR model can be simplified as

$$Y=\rho WY+Z\Theta +\varepsilon ,\varepsilon ∼N(0,\sigma^{2}I)$$ (3)

We will discuss Bayesian estimation method for model (3) in the next section.

## Bayesian estimation

We first build the Bayesian analysis framework of model (3) before giving a specific estimation step.

Given (*γ*,**c**), let **A** = (**I**—*ρ***W**), then the likelihood function of model (3) is

$$L(Y|\rho ,\Theta ,\gamma ,c,\sigma^{2})\propto \sigma^{\;-\;NT}\left|A\right|\text{exp}\left\{\;-\;\frac{1}{2\sigma^{2}}(A\text{Y}-\text{Z}\Theta {)}^{\prime }(A\text{Y}-\text{Z}\Theta )\right\}$$ (4)

The prior distribution of parameter *ρ* is usually assumed to be a uniform distribution with probability density function $\pi (\rho )=\frac{1}{\lambda_{\text{max}}^{-1}-\lambda_{\text{min}}^{-1}}$, where *λ*_max_, *λ*_min_ are the maximum and minimum eigenvalue of spatial weight matrix **W** respectively, which indicates that $\rho ∼U(\lambda_{\text{min}}^{-1},\lambda_{\text{max}}^{-1})$. Prior distribution of parameter **Θ** is set to be multiple normal distribution *N*(**μ**_0_, **Σ**_0_), where **μ**_0_ and **Σ**_0_ are the prior expectation and covariance. We also assume prior distribution of parameter *σ*^2^ as inverse gamma distribution *IG*(*α*,*β*) and set prior of *γ* and **c** as gamma distribution and normal distribution, that is *γ* ∼ *G* (*a*,*b*), **c** ∼ *N* (**μ**_*c*._**Σ**_*c*_).

Combining all the priors with likelihood function, we can obtain the joint distribution of all variables as follows

$$\text{P}(Y,\rho ,\Theta ,\gamma ,c,\sigma^{2})=L(Y|\rho ,\Theta ,\gamma ,c,\sigma^{2})\cdot \pi (\rho )\cdot \pi (\Theta )\cdot \pi (\gamma )\cdot \pi (c)\cdot \pi (\sigma^{2})$$ (5)

where *π*(·) denotes prior probability density function of each parameter. According to Bayesian theorem, the joint posterior distribution of all parameters is given by

$$\text{P}(\rho ,\Theta ,\gamma ,c,\sigma^{2})≜\text{P}(\rho ,\Theta ,\gamma ,c,\sigma^{2}|Y)$$ (6)

On the basis of joint distribution and joint posterior distribution, we can get the conditional posterior distribution of each parameter as follows

$$\text{P}(\Theta |\rho ,\gamma ,c,\sigma^{2})\propto \text{N}\left(\mu ,\Sigma \right)$$ (7)

where $\mu \;\text{=}\;{\left(Z^{\prime }Z\text{+}\sigma^{2}\Sigma_{0}^{\text{-}1}\right)}^{\text{-}1}(Z^{\prime }AY\text{+}\sigma^{2}\Sigma_{0}^{\text{-}1}\mu_{0})$ and $\Sigma \;\text{=}\;\sigma^{2}{\left(Z^{\prime }Z\text{+}\sigma^{2}\Sigma_{0}^{\text{-}1}\right)}^{\text{-}1}$. It can be seen from equation (7) that the conditional posterior distribution of **Θ** is multiple normal distribution when given other parameters. Similarly, the conditional posterior distributions of other parameters are as follows

$$\text{P}(\sigma^{2}|\rho ,\Theta ,\gamma ,c)\propto \text{IG}(\frac{\text{NT}}{2}\text{+}\alpha ,\frac{\left(AY\;\;-\;Z\Theta {)}^{\prime }(AY\;-\;Z\Theta \right)}{2}\text{+}\beta )$$ (8)

$$\begin{array}{l} \text{P}(\rho |\Theta ,\gamma ,c,\sigma^{2})\\ \propto \left|A(\rho )\right|\text{exp}\left\{\;-\;\frac{1}{2\sigma^{2}}(A(\rho )Y-Z\Theta {)}^{\prime }(A(\rho )Y-Z\Theta )\right\}\cdot \frac{1}{\lambda_{\text{max}}^{-1}-\lambda_{\text{min}}^{-1}} \end{array}$$ (9)

$$\text{P}(\gamma ,c|\Theta ,\rho ,\sigma^{2})\propto \exp\left\{-\frac{1}{2\sigma^{2}}(AY-Z\Theta {)}^{\prime }(AY-Z\Theta )\right\}\cdot \pi (\gamma )\cdot \pi (c)$$ (10)

where **A**(*ρ*) = (**I**—*ρ***W**). From the conditional posterior distributions of all parameters, we can see that the probability density functions of *γ*,**c** and *ρ* are more complex, and these parameters cannot be directly sampled. Therefore, we use Metropolis-Hastings algorithm to deal with this problem.

Assuming that the current value of *ρ* is *ρ*_*t*_ that meets P(*ρ*_*t*_|**Θ**,*γ*,**c**,*σ*^2^) > 0, and the candidate value ρ* is generated from the proposed distribution F(ρ*|ρ_*t*_) = *f* (ρ*—ρ), where *f* (·) is the probability density function, the transfer process is ρ* = ρ_*t*_ + *λz* where *z* ∼ *N*(0,1) and *λ* is a transfer parameter. Then the reception ratio of ρ* is *A*_1_(ρ*|ρ_*t*_) = min{1,*R*_1_}, where

$$R_{1}=\frac{\text{P}(\rho^{*}|\Theta ,\gamma ,c,\sigma^{2})F(\rho_{t}|\rho^{*})}{\text{P}(\rho_{t}|\Theta ,\gamma ,c,\sigma^{2})F(\rho^{*}|\rho_{t})}$$ (11)

Similarly, assuming that the current value of (*γ*,**c**) are (*γ*_*t*_,**c**_*t*_), and the candidate value (γ*,c*) are generated from the proposed distribution $\gamma^{*}∼N(\gamma_{t},\sigma_{\gamma }^{2})$ and $c^{*}∼N(c_{t},\sigma_{c}^{2}I)$ respectively. Then the reception ratio of (γ*,c*) is *A*_2_((γ*,c*)|(*γ*_*t*_,**c**_*t*_)) = min{1,*R*_2_}, where

$$R_{2}=\frac{\text{P}((\gamma^{*},c^{*})|\rho ,\Theta ,\sigma^{2})f_{\gamma }(\gamma_{t}|\left(\gamma^{*},\sigma_{\gamma }^{2}\right))f_{c}(c_{t}|\left(c^{*},\sigma_{c}^{2}\right))}{\text{P}((\gamma_{t},c_{t})|\rho ,\Theta ,\sigma^{2})f_{\gamma }(\gamma^{*}|\left(\gamma_{t},\sigma_{\gamma }^{2}\right))f_{c}(c^{*}|\left(c_{t},\sigma_{c}^{2}\right))}$$ (12)

$f_{\gamma }(\gamma_{t}|\left(\gamma^{*},\sigma_{\gamma }^{2}\right))$ represents the normal distribution probability density function of *γ*_*t*_ with mathematical expectation γ* and variance $\sigma_{\gamma }^{2}$. $f_{c}(c_{t}|\left(c^{*},\sigma_{c}^{2}\right))$ denotes the normal distribution probability density function of **c**_*t*_ with mathematical expectation c* and variance $\sigma_{c}^{2}$. $\sigma_{c}^{2}$ and $\sigma_{\gamma }^{2}$ are adjustment parameters. Z* and **Z**_t_ indicate the value of **Z** at corresponding time when the value of (*γ*,**c**) are (γ*,c*) and (*γ*_*t*_,**c**_*t*_)respectively.

We firstly employ Gibbs sampling method to generate parameters **Θ** and *σ*^2^ based on their conditional posterior distributions. Then we sample parameters *ρ*,*γ* and ***c*** by using Metropolis-Hastings algorithm. Specifically, the Bayesian estimation procedure of SLPSTR model is as follows.

1. Set the initial values of parameters (*ρ*,**Θ**,*γ*,***c***,*σ*^2^) as $\left(\rho_{0},\Theta_{0},\gamma_{0},c_{0},\sigma_{0}^{2}\right)$, let $\left(\rho_{t},\Theta_{t},\gamma_{t},c_{t},\sigma_{t}^{2}\right)$ be the results of *t*—*th* sampling;
2. Sample **Θ**_*t + 1*_ from the conditional distribution $\text{P}(\Theta |\rho_{t},\gamma_{t},c_{t},\sigma_{t}^{2})$;
3. Sample $\sigma_{t\text{+}1}^{2}$ from the conditional distribution P(*σ*^*2*^|*ρ*_*t*_, *γ*_*t*_, **c**_*t*_, **Θ**_t+1_);
4. Generate random number *r* from uniform distribution *U*(0,1) firstly, and then generate (ρ*,γ*,c*) from the following random process: ρ* = ρ_*t*_ + *λz*, the normal distribution $N(\gamma_{t},\sigma_{\gamma }^{2})$ and the normal distribution $N(c_{t},\sigma_{c}^{2}I)$ respectively, based on which we obtain (*ρ*_*t + 1*,_ *γ*_*t + 1*,_ ***c***_*t + 1*_) defined as
  $$\rho_{t+1}=\left\{ \begin{array}{l} \begin{array}{cc} \rho^{*}, & \text{if}\;r<A_{1}=\text{min}\left\{1,R_{1}\right\} \end{array}\\ \begin{array}{cc} \rho_{t}, & \text{others} \end{array} \end{array}\right.$$ (13)
  $$(\gamma_{\text{t+1}},c_{t+1})=\left\{ \begin{array}{l} \begin{array}{cc} (\gamma^{*},c^{*}), & \text{if}\;r<A_{2}=\min\left\{1,R_{2}\right\} \end{array}\\ \begin{array}{cc} (\gamma_{\text{t}},c_{t}), & \text{others} \end{array} \end{array}\right.$$ (14)
5. Let *t* = *t* + 1 and repeat step (ii)—(iv) until convergence. The convergence criterion $\frac{\left\|\left(\rho_{t},\Theta_{t},\gamma_{t},c_{t},\sigma_{t}^{2}\right)\right\|}{\left\|\left(\rho_{t-1},\Theta_{t-1},\gamma_{t-1},c_{t-1},\sigma_{t-1}^{2}\right)\right\|}\leq a$ is used in the process of estimation, where ‖·‖ represent Euclidean norm and *a* is accuracy requirement.

## Simulation

In this section, we conduct a Monte Carlo simulation to investigate the performance of Bayesian approach under small sample. The data generating process (DGP) is given by

$$y_{it}=\rho {\left(WY\right)}_{it}+x_{it}\beta_{0}+x_{it}\beta_{1}g\left(q_{it};\gamma ,c\right)+\mu_{i}+\varepsilon_{it}$$

where g(*q*_*it*_; *γ*, *c*) = (1 + exp(-*γ*(*q*_*it*_—*c*)))^-1^, *ρ* = 0.75, *γ* = 1.5, c = 3.5, $\beta_{0}={\left(\beta_{01},\beta_{02}\right)}^{\prime }\;\text{=}\;\left(2,\sqrt{5}\right)\prime$, **β**_1_ = (*β*_11_,*β*_12_)′ = (4,6)′ and *ε*_*it*_ ∼ *N* (0,0.25). In order to investigate the effect of spatial structure of data on the results of Bayesian, the weight matrix in [13] (Case matrix) and the Rook weight matrix in [14] are used respectively.. Other parameters and variables in DGP are set as follows.

1. Fixed effect *μ*_*i*_ is generated from uniform distribution *U* [–1,1];
2. Transition variable *q*_*it*_ follows from uniform distribution *U* [–8,8];
3. Every independent variables in *x*_*it*_ is generated from uniform distribution *U* [–5,5];
4. Random disturbance term *ε*_*it*_ follows from normal distribution *N*(0,*σ*^*2*^), where *σ*^*2*^ = 0.25.
5. The sample size is set to be 400 under both two spatial weight matrixes. And the convergence criterion of algorithm used in each estimation is $\frac{\left\|\left(\rho_{t},\Theta_{t},\gamma_{t},c_{t},\sigma_{t}^{2}\right)\right\|}{\left\|\left(\rho_{t-1},\Theta_{t-1},\gamma_{t-1},c_{t-1},\sigma_{t-1}^{2}\right)\right\|}\leq 0.01$.

The super-parameters in the priors are set as follows:*μ*_*0*_ = **0**, *μ*_*c*_ = **0**,∑_0_ = 100 · **I**_0_, ∑_*c*_ = *N* · **I**_*c*_, *α* = *β* = 0.001, *a* = *b* = 0.01, *λ* = 0.5, $\sigma_{\gamma }^{2}\;\text{=}\;0.01$, $\sigma_{c}^{2}\;\text{=}\;0.01$, where *N* is the number of cross-sections, **I**_0_ and **I**_c_ are identity matrixes.

For each generated data set, we estimate (*ρ*,**Θ**,*γ*,**c**,*σ*^*2*^) by using the proposed Bayesian approach for 10000 replications. We take the first 2000 replications as a burn-in and calculate the averages and variances of the other 8000 replications as the estimate of posterior means and standard deviations of coefficients.

The simulation results are listed in Table 1 from which we can see that: firstly, under both types of spatial weight matrix, the posterior means of all parameters are very close to their true value and the standard deviation is very small. This indicates that the Bayesian estimation has high accuracy and robustness under small sample. Secondly, the estimation accuracy of spatial correlation coefficient under Case matrix is higher than Rook matrix, which suggests that the estimation accuracy of the spatial correlation coefficient will decreases with the increase of spatial complexity of data. However, the estimation results of other coefficients are quite close under two weight matrices, indicating that the Bayesian method is applicable to spatial data with different complexity.

**Table 1:** The simulation results of parameters.

Coefficient	True value	Posterior Mean		Standard Deviation	
		Case	Rook	Case	Rook
*ρ*	0.75	0.7500	0.7499	0.0023	0.0032
*β*_01_	2	1.9912	1.9912	0.0233	0.0232
*β*_02_	$\sqrt{5}$	2.2220	2.2221	0.0240	0.0239
*β*_11_	4	4.0451	4.0451	0.0524	0.0523
*β*_12_	6	6.0691	6.0689	0.0588	0.0588
*γ*	1.5	1.3910	1.3913	0.0479	0.0478
*c*	3.5	3.5143	3.5143	0.0279	0.0278
*σ*^*2*^	0.25	0.2685	0.2682	0.0409	0.0409

**Fig 1:**
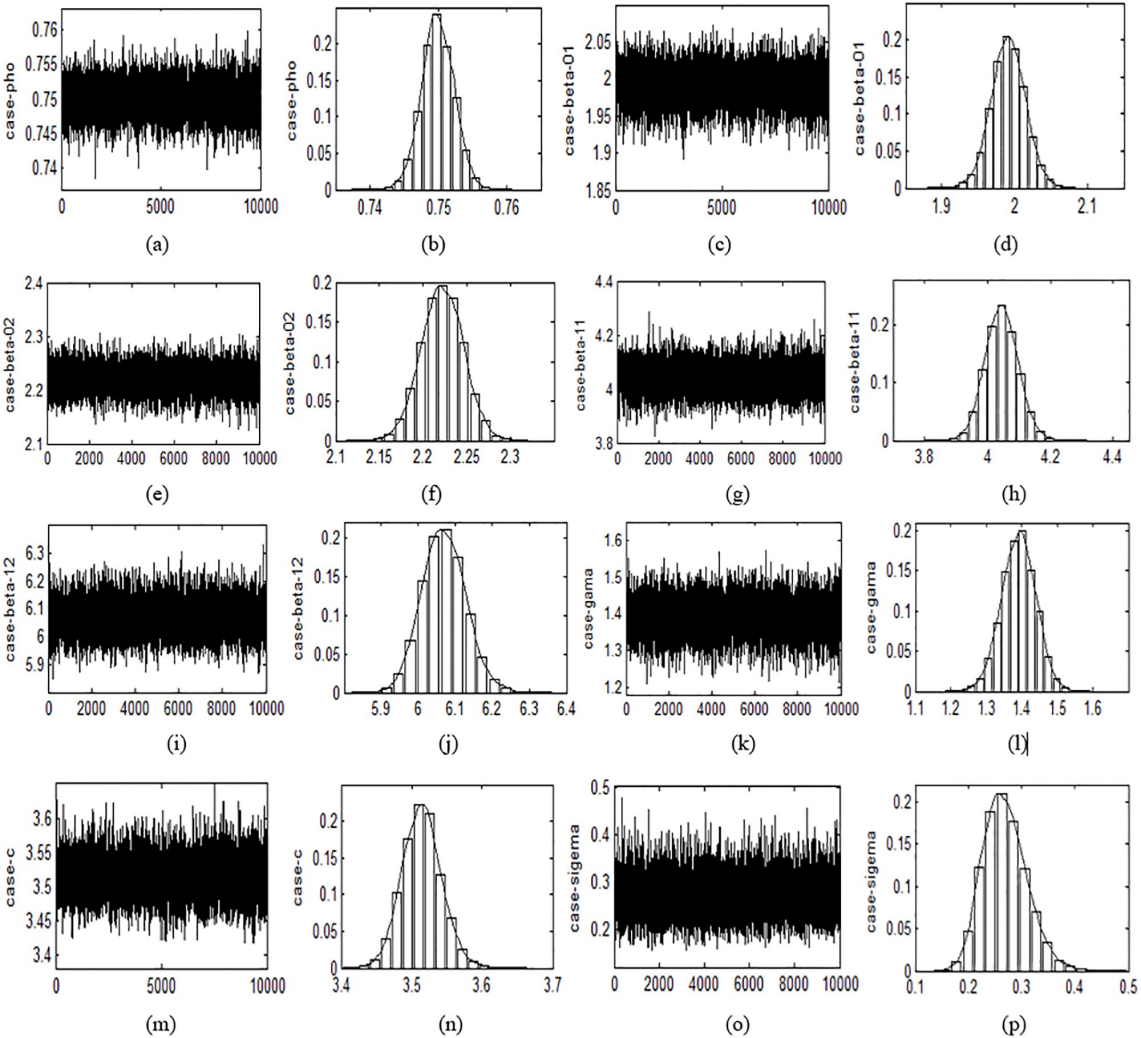
The sample track (left) and the posterior distribution frequency graph (right) of each parameter under the Case weight matrix.

**Fig 2:**
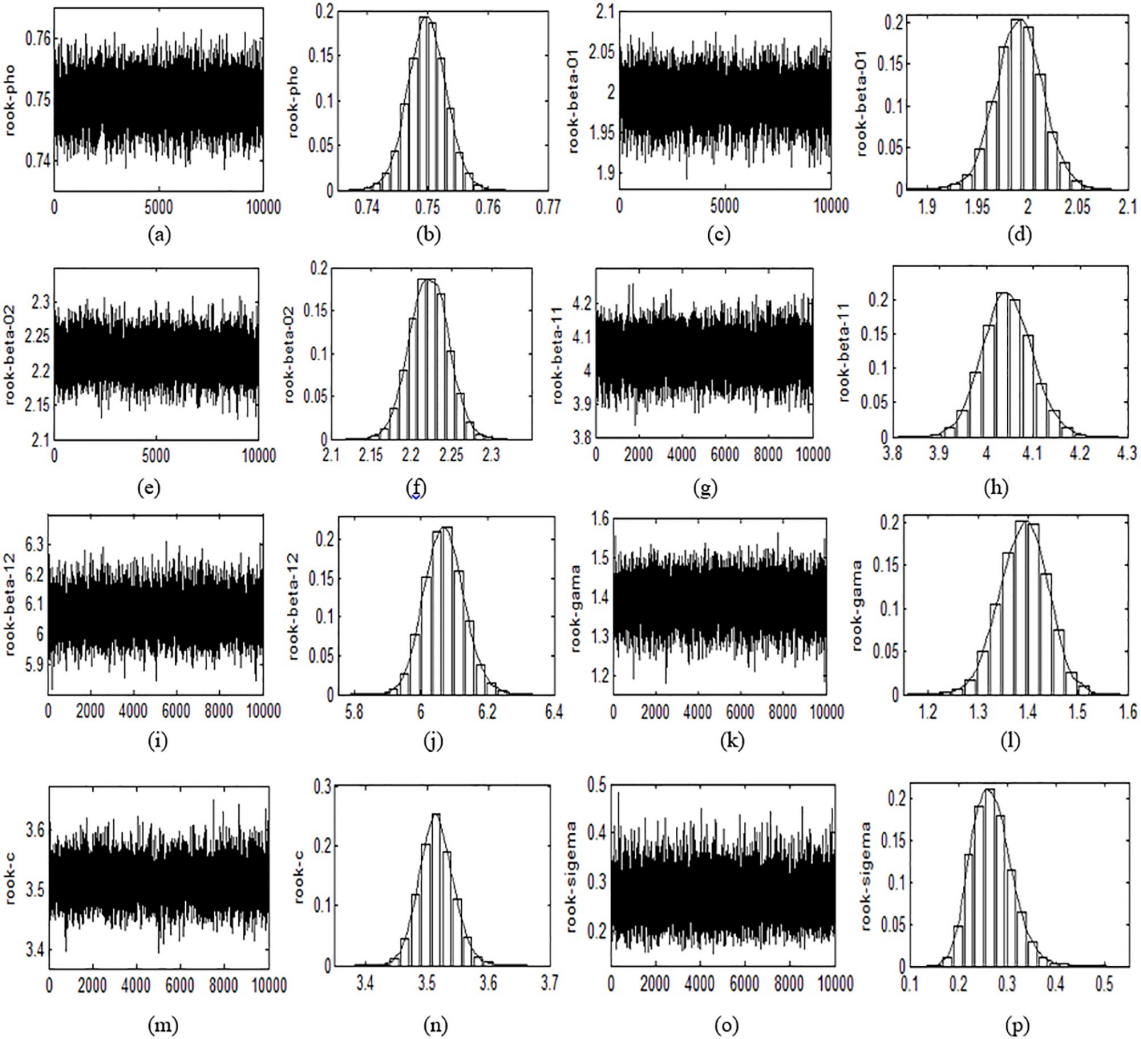
The sample track (left) and the posterior distribution frequency graph (right) of each parameter under the Rook weight matrix.

The sample track and frequency graph of posterior distribution for each coefficients based on 10000 replications are provided in Figs 1 and 2 respectively. We can see from the track graphs in Figs 1 and 2 that the track of each coefficient fluctuates around its true value under both Case weight matrix and Rook weight matrix. The amplitudes of fluctuation and deviations from true value of all coefficients are quite small, which shows that the accuracy and robustness of coefficient estimation are high. This further confirms the well performance of Bayesian approach under finite sample. The frequency graphs in Figs 1 and 2 show that most estimated values of 10,000 replications are located near their true values, which once again indicates that the proposed Bayesian approach has high estimation accuracy. It also can be seen from the two figures that the track and frequency distributions of each coefficient under different weight matrices are similar to each other, suggesting that the structure of spatial data has little effect on the estimation results. This reflects that the Bayesian method is applicable to a wide range of spatial data.

## A real data example

In this section, we apply our SLPSTR model and Bayesian estimation method to explore the impact of economic structure on economic growth, which has been a hot topic in the field of economic development (see [15], [16], [17], [18], [19]). Different from the existing literature, our study is based on the perspective of return to factors. Note that economic growth depends on the level of economic productivity which will ultimately reflect in the productivity level of factor that can be measured by the output elasticity of factor. This indicates that economic structure will affect economic growth through output elasticity of factor, so we take the following Cobb-Douglas aggregate production function as the basic model to conduct our empirical test:

lnY=αlnK+βlnL

where *Y*, *K* and *L* are output, capital stock and labor force, *α*,*β* represent output elasticity of capital and labor respectively.

Considering that China's regional economic development is not balanced and there exists wide range of spatial correlation between regional economies, we introduce the smooth transition nonlinear structure and spatial dependence into the basic model above and obtain the SLPSTR model as follows.

$$y_{it}=\rho {\left(WY\right)}_{it}+(\alpha_{1}k_{it}+\beta_{1}l_{it})+({\alpha^{\prime }}_{1}k_{it}+{\beta^{\prime }}_{1}l_{it})g\left(q_{it}^{\left(1\right)};\gamma_{1},c_{1}\right)+\mu_{i}+\varepsilon_{it}$$ (I)

$$y_{it}=\rho {\left(WY\right)}_{it}+(\alpha_{2}k_{it}+\beta_{2}l_{it})+({\alpha^{\prime }}_{2}k_{it}+{\beta^{\prime }}_{2}l_{it})g\left(q_{it}^{\left(2\right)};\gamma_{2},c_{2}\right)+\mu_{i}+\varepsilon_{it}$$ (II)

where *γ*_*it*_ = ln*Y*_*it*_, *k*_*it*_ = ln*K*_*it*_, *l*_*it*_ = ln*L*_*it*_. $q_{it}^{\left(1\right)}$ and $q_{it}^{\left(2\right)}$ are the transition variables refer to share of second industry and tertiary industry in GDP respectively. In model (I) (similar in model (II)), if the link function g(·) = 0, then we have *y*_*it*_ = *ρ*(**WY**)_*it*_ + (*α*_1_*k*_*it*_ + *β*_1_*l*_*it*_) + *μ*_*i*_ +*ε*_*it*_, under this circumstances we say economic growth is in a low regime. In contrast, we have $y_{it}=\rho {\left(WY\right)}_{it}+(\alpha_{1}+{\alpha^{\prime }}_{1})k_{it}+(\beta_{1}+{\beta^{\prime }}_{1})l_{it}+\mu_{i}+\varepsilon_{it}$ when g(·) = 1, and the economic growth is in a high regime here. With the change of transition variable $q_{it}^{\left(1\right)}$, the economic growth will transit from the low regime to the high regime continuously. Therefore, model (I) and model (II) can well describe the impact of economic structure on economic growth from the perspective of factor return. In addition, the introduction of spatial lag term (**WY**)_*it*_ can help to control the spatial convergence of regional economy, which will enhance the robustness of estimation results.

We take 281 cities in China as our study samples, and the raw data used in this study are from China city statistical yearbook 2007–2011. As to variable measure, firstly, we use GDP of city *i* in year *t* as a measure of total output *Y*_*it*_, and the GDP growth index of each city is apply as a deflator to eliminate the impact of price. Secondly, the labor force *L*_*it*_ is measured by the employment population of city *i* in year *t*. Thirdly, we use the proportion of secondary and tertiary industry in GDP of city *i* in year *t* as the measure of economic structure $q_{it}^{\left(1\right)}$ and $q_{it}^{\left(2\right)}$, respectively. Finally, the perpetual inventory method proposed by [20] is used to calculate the capital stock *K*_*it*_.

We next use the Bayesian estimation approach proposed in section 3 to estimate the empirical model (I) and (II). The estimation results are listed in Table 2. As shown in Table 2, the spatial correlation coefficients of model (I) and (II).are both positive, indicating that there exists positive spatial spillover effect in inter-city economy. The estimation results of Model (I) show that the smoothing parameter is 3.5589, which means that the speed of regime transition is slow and the transition between low and high regimes is continuous. In addition, the coefficients in linear part of model (I) are both positive, indicating that both capital and labor have a significant positive linear effect on economic growth. The coefficient of labor in non-linear part is positive and bigger than that in linear part, which suggests that the labor factor has a stronger positive nonlinear effect on economic growth. The result of capital stock is exactly the opposite of labor. The linear effect of capital stock on economic growth is much more significant than nonlinear effect. Moreover, the threshold of transition variable is 0.4247. This means that the regime transition occurs when the proportion of secondary industry reaches 42.47%.

**Table 2:** Estimation results of model (I) and model (II).

Variable	Model (I)		variable	Model (II)	
	Posterior Mean	Standard Deviation		Posterior Mean	Standard Deviation
*ρ*	0.3764	0.0219	*ρ*	0.5741	0.0624
*α*_1_	0.6817	0.0157	*α*_2_	0.4304	0.0523
*β*_1_	0.2211	0.0468	*β*_2_	0.9134	0.1514
${\alpha^{\prime }}_{1}$	0.0360	0.0377	${\alpha^{\prime }}_{2}$	0.3680	0.0628
${\beta^{\prime }}_{1}$	0.2572	0.1143	${\beta^{\prime }}_{2}$	-0.8090	0.1828
*γ*_1_	3.5589	0.5791	*γ*_2_	6.5124	0.7443
*c*_1_	0.4247	0.1383	*c*_*2*_	0.0876	0.2983

The estimation result of model (II) shows that the smoothing parameter is 6.5124, far greater than that of model (I). This result suggests that the speed of transition in model (II) is faster than model (I). Similar to model (I), the coefficients in linear part of model (II) are positive, this means that the linear effects of capital stock and labor on economic growth are both positive. The coefficient of the capital stock in nonlinear part of model (II) is positive and slightly smaller than that in linear part, indicating that the capital stock has a significant non-linear positive influence on economic growth. Furthermore, the coefficient of labor in non-linear part model (II) is negative, which indicates that the labor factor has a nonlinear negative effect on economic growth. Finally, we can see that the threshold of transition variable is 0.0876, suggesting that the regime transition begins when the proportion of tertiary industry is 8.76%, which is much smaller than the share of secondary industry, all this indicates that the impact of the tertiary industry is more important.

## Conclusion

In this paper, we propose a spatial lag panel smoothing transition model by introducing cross-section correlation of dependent variable into panel smoothing transition model and develop a Bayesian estimation approach for this model. In Bayesian analysis, Metropolis-Hastings algorithm and Gibbs method are used for sample design based on prior setting of each parameter in the model. A simulation study and real data estimation are conducted to investigate the practical effect of the SLPSTR model.. The numerical simulation results show that the proposed Bayesian method can perform well for a wide range of spatial data under infinite sample. The real data estimation results demonstrate the application value of the theoretical methods proposed in this paper.

## Supporting information

S1 Data Data used in real data study.xls. (XLS)

## References

[1] Hansen BE. Threshold Effects in Non-dynamic Panels: Estimation, Testing, and Inference. Journal of Econometrics. 1999, 93(2):345–368.

[2] Gonzalez A, Terasvirta T, Dijk DV. Panel Smooth Transition Regression Model and An Application to Investment Under Credit Constraints. Working Paper series in Economics and Finance 604, 2004.

[3] Gonzalez A, Terasvirta T, Dijk DV. Panel Smooth Transition Regression Models. Working papers, 2005.

[4] Fouquau J, Hurlin C, Rabaud I. The Feldstein-Horioka Puzzle: A Panel Smooth Transition Regression Approach. Economic Modelling. 2007, 25(2):284–299.

[5] Lesage JP. Bayesian Estimation of Spatial Autoregressive Models. International Regional Science Review. 1997, 20(1–2):113–129.

[6] Lesage JP. Bayesian Estimation of Limited Dependent Variable Spatial Autoregressive Models. Geographical Analysis. 2000, 32(1):19–35.

[7] Smith TE, Lesage JP. A Bayesian Probit Model with Spatial Dependencies. Advances in Econometrics. 2004, 18(18):127–160.

[8] Gelfand AE, Kottas A, MacEachern SN. Bayesian Nonparametric Spatial Modeling with Dirichlet Process Mixing. Journal of the American Statistical Association. 2005, 100(471): 1021–1035.

[9] Han X, Lee LF. Bayesian Estimation and Model Selection for Spatial Durbin Error Model with Finite Distributed Lags. Regional Science & Urban Economics. 2013, 43(5):816–837.

[10] Kang EL, Cressie N. Bayesian Inference for the Spatial Random Effects Model. Publications of the American Statistical Association. 2011, 106(495):972–983.

[11] Lesage JP. Spatial Econometric Panel Data Model Specification: A Bayesian Approach. Social Science Electronic Publishing. 2014, 9:122–145.

[12] Doğan O, Taşpınar S. Spatial Autoregressive Models with Unknown Heteroskedasticity: A Comparison of Bayesian and Robust GMM Approach. Regional Science & Urban Economics. 2014, 45(2):1–21.

[13] Case AC. Spatial Patterns in Household Demand. Econometrica. 1991, 59(4): 953–965.

[14] Su L. Semiparametric GMM Estimation of Spatial Autoregressive Models. Journal of Econometrics. 2012, 167(2):543–560.

[15] Carree MA. Technological Progress, Structural Change and Productivity Growth: a Comment. Structural Change and Economic Dynamics. 2003,14(1):109–115.

[16] Singh L. Technological Progress, Structural Change and Productivity Growth in the Manufacturing Sector of South Korea. World Review of Science, Technology and Sustainable Development. 2004, 1(1): 37–49.

[17] Ngai LR, Pissarides CA. Structural Change in a Multisector Model of Growth. American Economic Review. 2007, 97 (1): 429–443.

[18] Foellmi R, Zweimüller J. Structural Change, Engel’s Consumption Cycles and Kaldor’s Facts of Economic Growth. Journal of Monetary Economics. 2008, 55(7): 1317–1328.

[19] Timmer MP, de Vries GJ. Structural Change and Growth Accelerations in Asia and Latin America: A New Sectoral Data Set. Cliometrica. 2009, 3(2): 165–190.

[20] Zhang J, Wu GY, Zhang JP. The Estimation of China’s Provincial Capital Stock: 1952–2000. Economic Research Journal. 2004, (10): 35–44.

